# Retinal regeneration in the *Xenopus laevis* tadpole: a new model system

**Published:** 2009-05-18

**Authors:** M. Natalia Vergara, Katia Del Rio-Tsonis

**Affiliations:** Department of Zoology, Miami University, Oxford, OH

## Abstract

**Purpose:**

Retinal regeneration research holds potential for providing new avenues for the treatment of degenerative diseases of the retina. Various animal models have been used to study retinal regeneration over the years, providing insights into different aspects of this process. However the mechanisms that drive this important phenomenon remain to be fully elucidated. In the present study, we introduce and characterize a new model system for retinal regeneration research that uses the tadpole of the African clawed frog, *Xenopus laevis*.

**Methods:**

The neural retina was surgically removed from *Xenopus laevis* tadpoles at stages 51–54, and a heparin-coated bead soaked in fibroblast growth factor 2 (FGF-2) was introduced in the eyes to induce regeneration. Histological and immunohistochemical analyses as well as DiI tracing were performed to characterize the regenerate. A similar surgical approach but with concomitant removal of the anterior portion of the eye was used to assess the capacity of the retinal pigmented epithelium (RPE) to regenerate a retina. Immunohistochemistry for FGF receptors 1 and 2 and phosphorylated extracellular signal-regulated protein kinase (pERK) was performed to start elucidating the intracellular mechanisms involved in this process. The role of the mitogen activated protein kinase (MAPK) pathway was confirmed through a pharmacological approach using the MAPK kinase (MEK) inhibitor U0126.

**Results:**

We observed that *Xenopus laevis* tadpoles were able to regenerate a neural retina upon induction with FGF-2 in vivo. The regenerated tissue has the characteristics of a differentiated retina, as assessed by the presence and distribution of different retinal cell markers, and DiI tracing indicated that it is able to form an optic nerve. We also showed that retinal regeneration in this system could take place independently of the presence of the anterior eye tissues. Finally, we demonstrated that FGF-2 treatment induces ERK phosphorylation in the pigmented epithelia 10 days after retinectomy, and that inhibition of the MAPK pathway significantly decreases the amount of retina regenerated at 30 days post-operation.

**Conclusions:**

Regeneration of a complete neural retina can be achieved in larval *Xenopus laevis* through activation of the MAPK signaling pathway by administering exogenous FGF-2. This mechanism is conserved in other animal models, which can regenerate their retina via pigmented epithelium transdifferentiation. Our results provide an alternative approach to retinal regeneration studies, capitalizing on the advantages of the *Xenopus laevis* tadpole as a model system.

## Introduction

Millions of people worldwide are affected by retinal degenerative diseases, such as macular degeneration, diabetic retinopathy, or glaucoma, that lead to vision loss or blindness. To date, even though treatment is often promising at early stages of these diseases, once the retina is severely damaged there is no possibility for functional recovery. In this context, retinal regeneration research could lead to the development of new therapeutic strategies for the treatment of these pathologies.

Certain urodele amphibians represent ideal animal models for this type of studies due to their outstanding regenerative capacity. A great deal of progress has been made in recent years toward the understanding of the molecular mechanisms that drive spontaneous retinal regeneration in these animals [1-7]. In this system, the retinal pigmented epithelium (RPE) is able to regenerate an injured or lost neural retina through a process of transdifferentiation, which involves the dedifferentiation of these mature cells, their proliferation, and subsequent differentiation into all the various cell types that constitute the normal tissue. The implications of understanding this phenomenon are evident, yet the lack of molecular biology tools to work with these urodele amphibians coupled with their large and unsequenced genome constitute difficulties in their use as animal models. Thus other vertebrate model organisms have been established for retinal regeneration research. One good example is the embryonic chick model, which is starting to provide insights into the molecular pathways involved in retinal regeneration from different cellular sources in the eye including RPE transdifferentiation [8,9]. However, despite the clear advantages of this model, chicks are only able to regenerate their entire retina during a small window of embryonic development when the neural retina is not yet fully differentiated [8,9]. Other animal models, such as adult goldfish and zebrafish, have been established as well, in which Müller glia cells have been demonstrated to have the potential to regenerate retinal neurons [10-15]. However this can only take place if the retinal loss is not complete and the process does not involve RPE transdifferentiation. The limitations of the available animal models prompted us to establish a new system to investigate the process of retinal regeneration through pigmented epithelium transdifferentiation.

*Xenopus laevis* is probably the most well studied anuran amphibian in laboratories. It has been used in the developmental biology field for a long time. Many of its genes have been identified, and a wide variety of molecular biology techniques have already been established for this species. However, its potential in retinal regeneration research has not been exploited to its fullest. The capacity of the *Xenopus laevis* RPE to transdifferentiate into neural retina has been demonstrated through the transplantation of RPE explants from the eyes of tadpoles or adult *Xenopus laevis* into the eyes of tadpoles that had been lentectomized [16,17]. Transdifferentiation of RPE into retina in this system requires the influence of certain factors provided by the neural retina, since explants transplanted into the orbit of an enucleated eye, as well as those transplanted into the anterior chamber of host eyes, failed to transdifferentiate. But what are the factors produced by the mature retina that induce such fate decisions in the RPE? Studies performed in culture using RPE explants from tadpoles suggest that a good candidate for such a molecule is fibroblast growth factor 2 (FGF-2), since incubation of the explants in the presence of this factor for up to 30 days induced their transdifferentiation in vitro into different retinal neuron and glial types [17]. FGF-2 has also been shown to be an induction factor in RPE transdifferentiation in other animal models such as the embryonic chick [8,9]. However there must be additional mechanisms in place that prevent the retina from inducing transdifferentiation of its normal adjacent RPE layer.

In vivo approaches to retinal regeneration in *Xenopus laevis* have, however, been scarce. Upon resection of up to two-thirds of the eye in tadpoles beginning at stage 32, a repair process occurs that can involve a round-up of the eye to close the wound, or the regeneration of the missing structures by proliferation and migration of the cells from the remaining tissues [18-22]. This system has been useful in the study of retinotectal connectivity patterns, but the regenerative process has not been characterized at the cellular and molecular level, and the mechanisms involved have not been elucidated. In adult *Xenopus laevis,* partial retinal regeneration through proliferation of cells in the ciliary margin of the eye has been observed [23]. In contrast, Yoshii et al. showed that when the retina is removed in postmetamorphic frogs, leaving behind both the RPE and the vascular membrane of the eye, RPE cells migrate and attach to the vascular membrane, where they are induced to transdifferentiate into neural retina [24]. This is a promising system to study RPE transdifferentiation; however, morphometric and molecular characterization of the regenerated retina still needs to be performed, and the inducing factors present in the vascular membrane and molecular mechanisms involved in the process have yet to be identified.

Considering its effects in vitro, FGF-2 seems to be a reasonable candidate to induce this process, yet no in vivo studies have attempted to exogenously administer this protein to retinectomized *Xenopus laevis* eyes. In the present work, we introduce a new model system to study retinal regeneration following complete neural retina removal (including the vascular membrane), using the tadpole of the African clawed frog, *Xenopus laevis*. We show that in these animals FGF-2 is able to induce regeneration of a complete retina in vivo, at a stage in which the eyes are already fully differentiated, having acquired the final structure and cell types that will be maintained in the adult. This constitutes an advantage over embryonic systems in which it is difficult to dissect developmental events from those exclusive to regeneration. In addition, there are technical and financial advantages to establishing a model for research using tadpoles as opposed to postmetamorphic frogs, among them: ease of obtaining and maintaining large numbers of animals in the laboratory; reduced costs from not needing large tank setups; and speed in procuring animals that are at the appropriate stage.

Moreover, we show that the regenerated retina is similar to the intact one in the expression of different differentiation markers and in other morphometric parameters, as well as in its ability to project axons to form an optic nerve. In addition, our data suggests that the RPE is likely to be the source of retinal regeneration in this system, and that upon retina removal, the expression of FGF receptors 1 and 2 is upregulated in the pigmented epithelium. We also show that regeneration in this model employs a mechanism that is common to other animal models and involves the activation of the mitogen activated protein kinase (MAPK) pathway, since FGF-2 induces phosphorylation of extracellular signal- regulated protein kinase (ERK) in the RPE, and inhibition of this pathway significantly reduces retinal regeneration. Our studies provide a new system to study the molecular mechanisms underlying retinal regeneration by exploiting the advantages of this well established animal model, and point to some of the important factors that control this process.

## Methods

### Animals, surgeries, and tissue processing

*Xenopus laevis* tadpoles were obtained from our own frog colony, following a protocol approved by the Institutional Animal Care and Use Committee. The adult frogs used for breeding were a kind gift from Dr. Donald Sakaguchi (Iowa State University, Ames, IA). The tadpoles were kept in tanks with 40% Holtfretter’s solution at 25 °C and were fed tadpole brittle (Nasco, Fort Atkinson, WI) until they reached stages 51–54. They were then anesthetized by immersion in 0.02% MS222 (Sigma, St. Louis, MO) diluted in 40% Holtfretter’s solution for 5 min, and placed on their side in a wax well to hold them during microsurgery. Only one eye was operated per animal to increase the survival rate. The cornea was cut with a scalpel and sharp forceps, and the lens was removed through the pupil with forceps. A pulled glass pipette was employed to gently blow Holtfretter’s solution inside the eye, which promoted the detachment of the retina. The neural retina was then easily removed, and a heparin-coated acrylic bead (Sigma) was placed inside the eye. Control animals received beads that had been soaked in PBS (3.2 mM Na_2_HPO_4_, 0.5 mM KH_2_PO_4_, 1.3 mM KCl, 135 mM NaCl, pH 7.4) for at least 2 h, and experimental animals received beads soaked for at least 2 h in a 0.5 µg/µl solution of FGF-2 (R&D Biosystems, Minneapolis, MN) in PBS. The animals were allowed to recover and were kept alive until they were collected at 0, 10, 15, 20, and 30 days post-surgery. At collection times the animals were euthanized by overexposure to anesthesia (1 h) followed by fixation.

For histological processing, tadpoles were fixed in 100% Bouin’s solution (Fisher Scientific, Pittsburgh, PA) and embedded in paraffin. Next, 12 µm sections were cut and stained with Harris’ hematoxylin and eosin Y (both from Fisher Chemical, Fairlawn, NJ). For immunohistochemistry, tadpoles were fixed in 4% formaldehyde in PBS for 4 h and washed several times in PBS. These were then placed in 30% sucrose at 4 °C overnight and embedded in optimal cutting temperature medium (OCT; Tissue-Tek, Sakura Finetek USA, Torrance, CA), to be frozen for cryosectioning. Intact stage 46 tadpoles were also collected and processed for immunohistochemistry for some experiments.

Only for experiments designed to test if the RPE is a source of regeneration did we take a different surgical approach: we anesthetized the animals and cut out the anterior third of the eye with micro scissors and discarded it. We then removed the neural retina with forceps, and used a stream of Holtfretter’s solution to rinse the eyecup. Either no bead, a control heparin bead, or an FGF-2-soaked heparin bead was placed in the eyecup, and the animals were allowed to recover. The eyes were collected at 30 days postsurgery, fixed in Bouin’s solution, and processed for histology.

### Immunohistochemistry

Cryosections, 10 µm thick, were rinsed three times in PBS for 5 min each time and then once in 0.5% PBS-Triton X-100 for 10 min, followed by another three washes in PBS. These were blocked in PBS containing 0.05% Triton X-100 and 1% BSA for 1 h at room temperature, washed in PBS, and incubated in 1% saponin in PBS for 5 min to permeabilize. After more PBS washes, sections were incubated overnight at 4 °C with the primary antibodies diluted in blocking solution. These were then washed three times for 10 min each in PBS and incubated in secondary antibody diluted in blocking solution for 2 h at room temperature. These were again washed in PBS before being mounted with Vectashield (Vector Laboratories, Burlingame, CA) and analyzed under a confocal microscope. The following mouse monoclonal primary antibodies were used at a 1:20 dilution: Xen-1 (developed by Dr. Ariel Ruiz I Altaba, University of Geneva Medical School, Geneva, Switzerland), 39.4D5 (anti-islet-1, developed by Dr. Thomas Jessell, Columbia University, New York, NY), 3B5 (anti-AP2α, developed by Dr. Trevor Williams, University of Colorado, Denver, CO), and Xap-2 (developed by Dr. Donald Sakaguchi, Iowa State University, Ames, IA, and by Dr. W.A. Harris, University of Cambridge, Cambridge, UK). These antibodies were obtained from the Developmental Studies Hybridoma Bank and were developed under the auspices of the National Institute of Child Health and Human Development and maintained by the University of Iowa, Department of Biologic Sciences (Iowa City, Iowa). Mouse anti RPE-65 was purchased from Chemicon (Millipore Corporation, Billerica, MA) and diluted 1:250. A goat anti-mouse antibody conjugated with Alexa Fluor 488 (Molecular Probes, Invitrogen, Carlsbad, CA), diluted 1:100, was used as a secondary antibody.

For H5 (anti-vimentin, developed by Dr. Joshua Sanes, Harvard University, Cambridge, MA, and obtained from Developmental Studies Hybridoma Bank) immunohistochemistry, sections were washed in PBS three times for 5 min each time and then once in 1% H_2_O_2_ in PBS for 5 min, followed by another three washes in PBS. A mouse ABC staining system (Santa Cruz Biotechnology, Santa Cruz, CA) was employed for color immunohistochemistry, following the manufacturer’s instructions, with a primary antibody concentration of 1:10 in the kit’s blocking solution.

For other antibodies, the protocol used was as follows: after three washes in PBS the slides were incubated for 5 min in 1% saponin in PBS, followed by another three PBS washes. The sections were then blocked with 10% goat serum diluted in 0.03% PBS-Triton X100, followed by an overnight incubation with primary antibody diluted in blocking solution at 4 °C. Mouse anti-diphosphoERK 1/2 was obtained from Sigma and used at a 1:100 dilution. Rabbit polyclonal anti-FGFR antibodies (flg, or FGFR1, and bek, or FGFR2) were purchased from Santa Cruz Biotechnology and used at a 1:20 dilution. Rabbit polyclonal anti-recoverin antibody (Chemicon, Millipore Corporation) was used at a 1:1,000 dilution. After three 15-min washes in PBS, the slides were incubated in the secondary antibody solution, goat anti-rabbit Alexa Fluor 546 (Molecular Probes, Invitrogen) diluted 1:100, for 2 h at room temperature, further washed in PBS and mounted with Vectashield.

### Cell counts and measurements

Specimens used for comparing cell numbers in each retinal cell layer were prepared as follows: 10 µm-thick cryosections of intact eyes and 30-day-postretinectomy eyes exposed to FGF-2 beads were rinsed in PBS and mounted using Vectashield with DAPI (Vector, Burlingame, CA) to fluorescently label nuclei. High magnification pictures (60X) were taken with an Olympus FV500 confocal microscope, and cell counting was performed using Image Pro Plus Software (Media Cybernetics, Bethesda, MD) on rectangular 50×100 μm areas (that is, 50 μm long and spanning the whole width of the retina). Twelve different sections corresponding to six different eyes from different tadpoles (two sections/different eye) were analyzed for each group, and the percentage of cells in each layer with respect to the total number of cells in each area was determined.

The length of rod outer segments was compared after preparing specimens as follows. Intact and 30-day regenerated tadpole eyes were embedded in paraffin, sectioned at 10 μm thickness, and stained with hematoxylin and eosin. Light microscopy pictures were taken at 40X magnification, and the length of rod outer segments was measured using Image Pro Plus Software. For each group, 232 rods were measured over three different sections each of six different eyes (a total of 18 sections evaluated). Microsoft Excel was used for statistical calculations, and a Student *t*-test was performed to assess significance.

### DiI labeling

Stages 51–54 intact *Xenopus laevis* tadpoles, as well as 30-day-postretinectomy tadpoles exposed to either FGF-2 or control-soaked beads, were anesthetized in MS222 and fixed in 4% formaldehyde in PBS for 6 h. A pulled glass capillary attached to a mouth pipette was used to inject 0.5 μl of 1, 1'-dioctadecyl-3,3,3′,3′-tetramethylindocarbocyanine perchlorate (DiI) solution (Molecular Probes, Invitrogen) inside the eyes. The tadpoles were then placed in PBS at 37 °C for 10 days. Whole-mount images were obtained with a camera attached to a fluorescence inverted microscope.

### Treatment with inhibitors

U0126 (Calbiochem, EMD Chemicals, Gibbstown, NJ), an inhibitor of MAPK kinase (MEK), was dissolved in DMSO to a concentration of 100 µM or 1 mM, and affigel blue beads (BioRad, Hercules, CA) were soaked in this solution for at least 2 h at room temperature. Control affigel blue beads were soaked in DMSO alone. Surgeries and collections were performed as described but one of these beads was introduced in the operated eyes in addition to the FGF-2 bead.

## Results

### FGF-2 induces retinal regeneration in the *Xenopus laevis* tadpole in vivo

**Figure 1:**
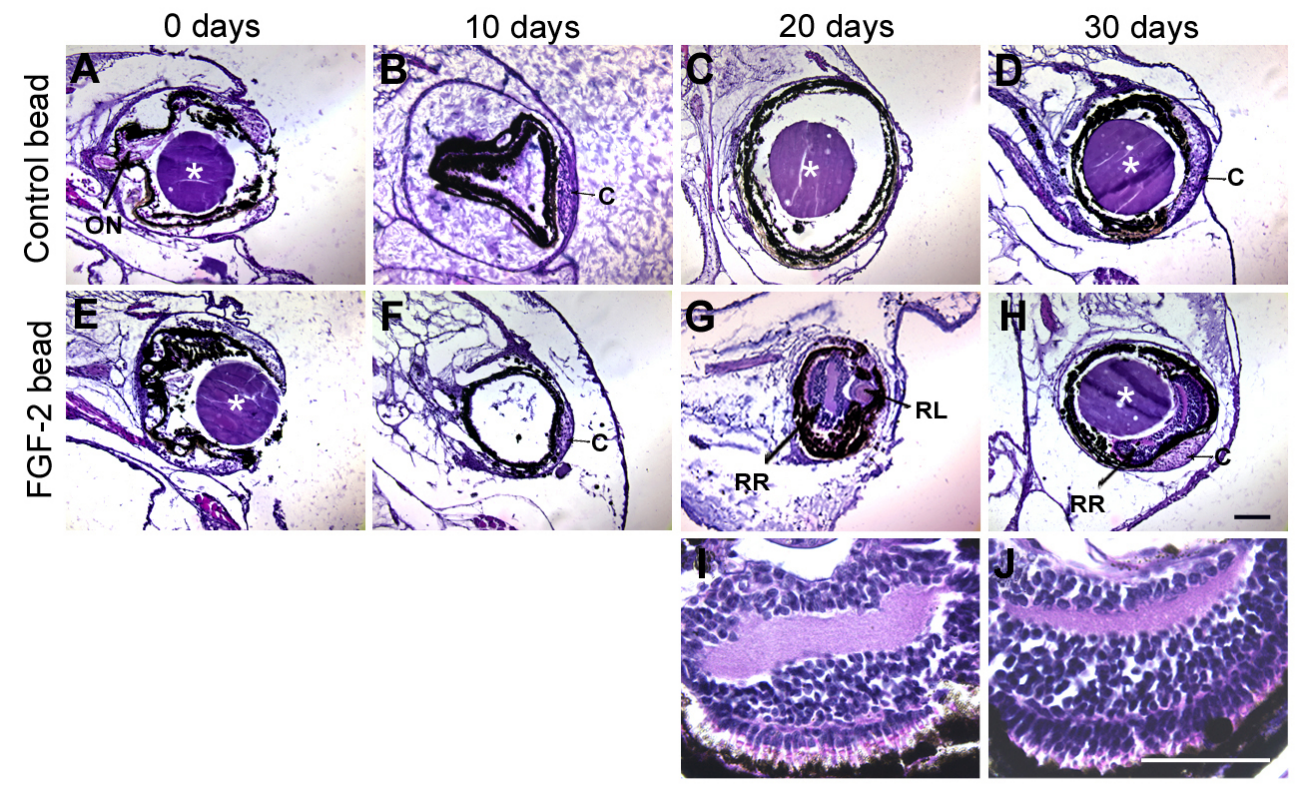
FGF-2 induces retinal regeneration in *Xenopus laevis* in vivo after complete retinectomy. Shown are sections of retinectomized tadpole eyes in which a control bead (**A**-**D**) or an FGF-2-soaked bead (**E**-**J**) was introduced at day 0. The eyes were collected at postoperative days 0 (**A** and **E**), 10 (**B** and **F**), 20 (**C**, **G**, **I**), and 30 (**D**, **H**, **J**), and stained with hematoxylin and eosin. Notice the absence of nonpigmented tissues inside the eye at the earlier time points. At 20 days, a layered neural retina was evident only in the eyes treated with FGF-2 (**G**); this retina was larger by 30 days (**H**). **I** and **J** are close up images of the regenerated retinas observed in **G** and **H** respectively. Abbreviations: optic nerve (ON); cornea (C); regenerated retina (RR); regenerated lens (RL). Asterisks indicate control or FGF-2-soaked bead. Scale bars represent 100 μm (scale bar in **H** applies to **A-H** and scale bar in **J** applies also to **I**).

Considering that FGF-2 had previously been used to induce transdifferentiation of *Xenopus laevis* tadpole RPE into neural retina in vitro [17], we decided to test its ability to induce retinal regeneration in vivo at a stage in which the eye is already fully differentiated. We surgically removed the whole neural retina as well as the ciliary margin and nonpigmented ciliary epithelium in stages 51–54 tadpoles. We then introduced either an FGF-2-soaked heparin bead or a control heparin bead soaked in vehicle solution in the operated eyes. The animals were euthanized at various times after surgery and processed either for histology (Figure 1) or for immunohistochemistry using antibodies against Xen1, which specifically labels neural tissue including the retina, and RPE-65, which labels the RPE (Appendix 1).

We found that FGF-2 alone was able to induce regeneration of a neural retina that seemed to have all three cellular layers by 20 days postretinectomy (Figure 1G,I Appendix 1, panel D), and was larger at 30 days (Figure 1H,J, Appendix 1, panel E). Even though the regenerated retina appeared histologically normal, it did not always form in the posterior part of the eye but was sometimes shifted anteriorly (Figure 1H and Appendix 1, panel E). No retinal regeneration was observed in the control eyes (Figure 1A-D). The source of the newly formed tissue cannot be identified by this experiment, but it is likely to be one of the pigmented tissues of the eye (RPE, pigmented ciliary body epithelium, or iris) since no nonpigmented tissues were left behind after surgery that could be identified by histological staining or by Xen1 immunohistochemistry (Figure 1A, Appendix 1, panel B). RPE-65 immunolabeling was observed only in the RPE at all times tested (Appendix 1, panel F-J). This is interesting because during newt retinal regeneration, which occurs through transdifferentiation of the RPE, this protein can be seen in the regenerating retina for up to 20 days postretinectomy [25]. However these results do not eliminate the possibility that the RPE might be the source of regeneration, since the physiology of frog tissues might be different from that of newts.

### Regenerated retina is properly differentiated and can form an optic nerve

**Figure 2:**
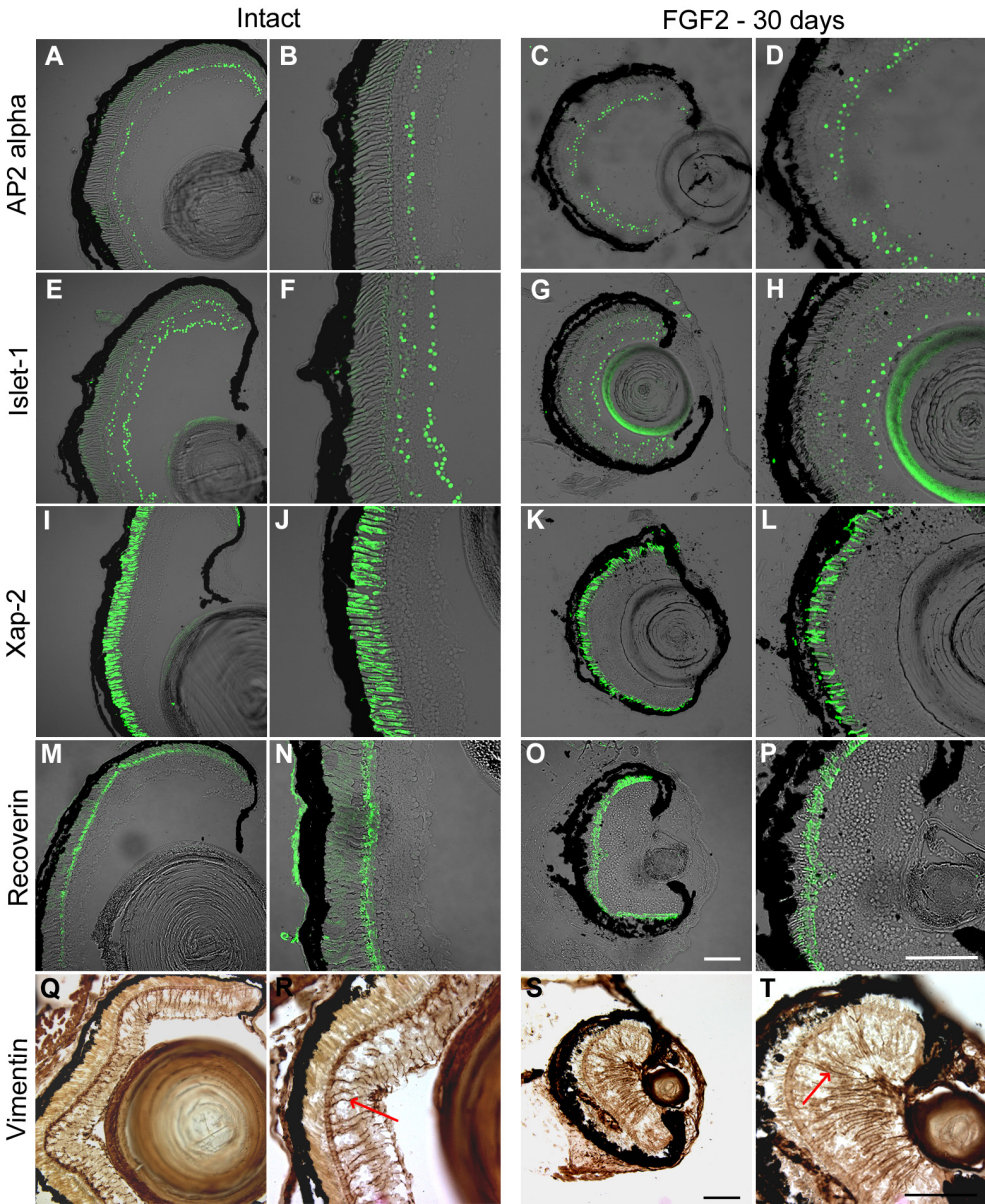
Regenerated retina induced by FGF-2 expresses markers of normal retinal cells. Intact eyes (**A**, **B**, **E**, **F**, **I**, **J**, **M**, **N**, **Q**, **R**) or eyes 30 days postretinectomy with FGF-2 administration (**C**, **D**, **G**, **H**, **K**, **L**, **O**, **P**, **S**, **T**) were immunolabeled for different retinal cell markers. **B, F, J, N, R, D, H, L, P,** and **T** are close up images of the retinas shown in **A**, **E**, **I**, **M**, **Q**, **C**, **G**, **K**, **O**, and **S**, respectively. **A**-**D**: AP2α was used as a marker for amacrine cells. **E**-**H**: Islet-1 was used as a marker of ganglion cells but could also detect some subpopulations of amacrine, bipolar, and horizontal cells. **I**-**L**: Xap-2 was used to mark rod photoreceptors. **M**-**P**: Recoverin was used to detect photoreceptors and midget cone bipolar cells. **Q**-**T**: Vimentin was used as a marker of Müller glia. Red arrows point at dark-colored Müller glia processes labeled with the vimentin antibody. There was a general light brown background throughout the sections, whereas the staining of the antibody was actually dark brown. All the markers tested were expressed in both the intact and regenerated retinas. Scale bars represent 100 μm (Scale bars in **O** and **S** apply to **A**, **E**, **I**, **M**, **Q**, **C**, **G**, **K**, **O**, **S**; scale bars in **P** and **T** apply to **B**, **F**, **J**, **N**, **R**, **D**, **H**, **L**, **P**, **T**).

Having established the ability of FGF-2 to induce retinal regeneration in this system, we went on to characterize the regenerated retina. To assess the presence and normal localization of the different cell types that constitute the normal retina in the regenerated tissue, we performed immunohistochemistry for different retinal cell markers on sections of eyes that had been retinectomized, treated with an FGF-2-soaked bead, and collected 30 days postsurgery. We used an antibody against AP2α, which has been shown to label amacrine cells and, more weakly, horizontal cells (Figure 2A-D) [26,27]. Islet-1 antibody was used to label mainly ganglion cells but it has also been reported to detect some subpopulations of amacrine, bipolar, and horizontal cells (Figure 2E-H) [27-29]. We used Xap-2 antibody to label rod photoreceptors (Figure 2I-L) [17,30]. Recoverin antibody was used to label photoreceptors and midget cone bipolar cells (Figure 2M-P) [31-33], and Vimentin antibody, was used to detect Müller glia cells (Figure 2Q-T) [34]. As expected, the regenerated retina possessed each cell type tested, located in a pattern similar to that of an intact retina. However, a slight difference was evident in the inner nuclear layer of these retinas since the regenerated ones showed a broader expression pattern of markers such as AP2α and islet-1 in this layer. Therefore we performed immunofluorescence for these markers on younger intact eyes (stage 46 tadpoles) to determine if the pattern observed in the regenerates corresponded to that of a differentiated but younger eye. The results, shown in Appendix 2, suggest that this is indeed the case.

To further characterize the regenerated retinas, we counted the nuclei in the different cell layers and compared these numbers to those of intact retinas. We found no significant difference in the percentage of cells in each layer between the two groups (Appendix 3). In addition, we did not find a significant difference in the length of the rod outer segments between intact and regenerated retinas (Appendix 3).

**Figure 3:**
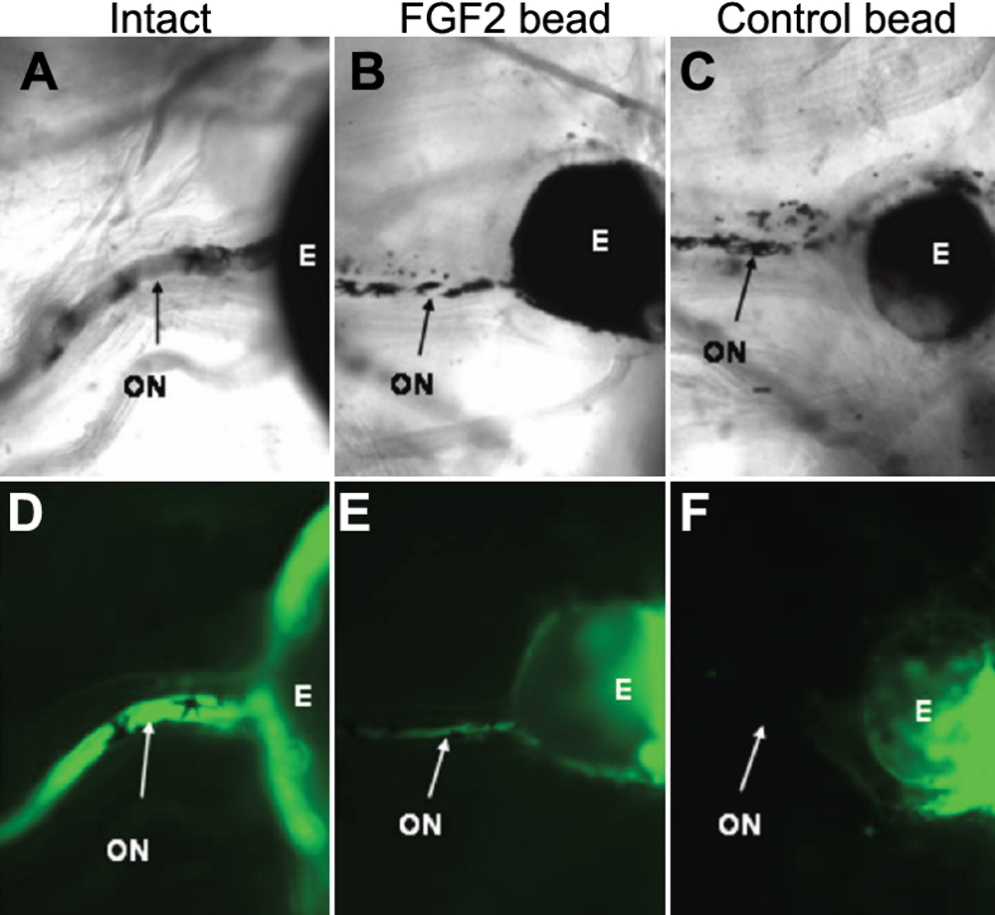
The regenerated retina is able to form an optic nerve. Intact eyes (**A**, **D**) and eyes 30 days postretinectomy exposed to either an FGF-2 bead (**B**, **E**) or a control bead (**C**, **F**) were injected with DiI to label cell membranes. Ten days later, the ganglion cell axons could be seen projecting through the optic nerve in both intact and FGF-2 exposed eyes (**D** and **E**). Control retinectomized eyes did not regenerate a retina and therefore did not project their axons through what remained of the optic nerve (**F**). **D**, **E**, and **F** correspond to fluorescent views (DiI labeling) of the bright-field images shown in **A**, **B**, and **C** respectively. Abbreviations: eyeball (E); optic nerve (ON).

Finally, we investigated if the regenerated retinas were able to project ganglion cell axons to form an optic nerve. We used the fluorescent lipophilic dye, DiI, which labels and spreads through cell membranes. We injected this dye in intact eyes, and eyes 30 days postretinectomy that had been exposed to control or FGF-2 soaked beads. Ten days later, fluorescent staining of the optic nerve in whole-mount preparations of tadpoles with intact eyes (Figure 3D) was an indicator that the ganglion cells were labeled by this tracer, which spread through their axons. This staining of the optic nerve was also evident in 6 out of 10 cases of retinectomized tadpoles exposed to FGF-2-soaked heparin beads (Figure 3E), an indication that the regenerated retinas were able to project axons to form an optic nerve. In contrast, no staining of the optic nerve was observed in any case (0 out of 10 cases) of retinectomized tadpoles exposed to control heparin beads (Figure 3F), since this treatment does not lead to regeneration of the neural retina. In Figure 3C, the optic nerve seen in the light microscopy image of control eyes is the remnant of the original one but does not contain axons, since their cell bodies were removed by the retinectomy procedure and therefore the axons degenerated.

### The RPE is likely a source of retinal regeneration in vivo

**Figure 4:**
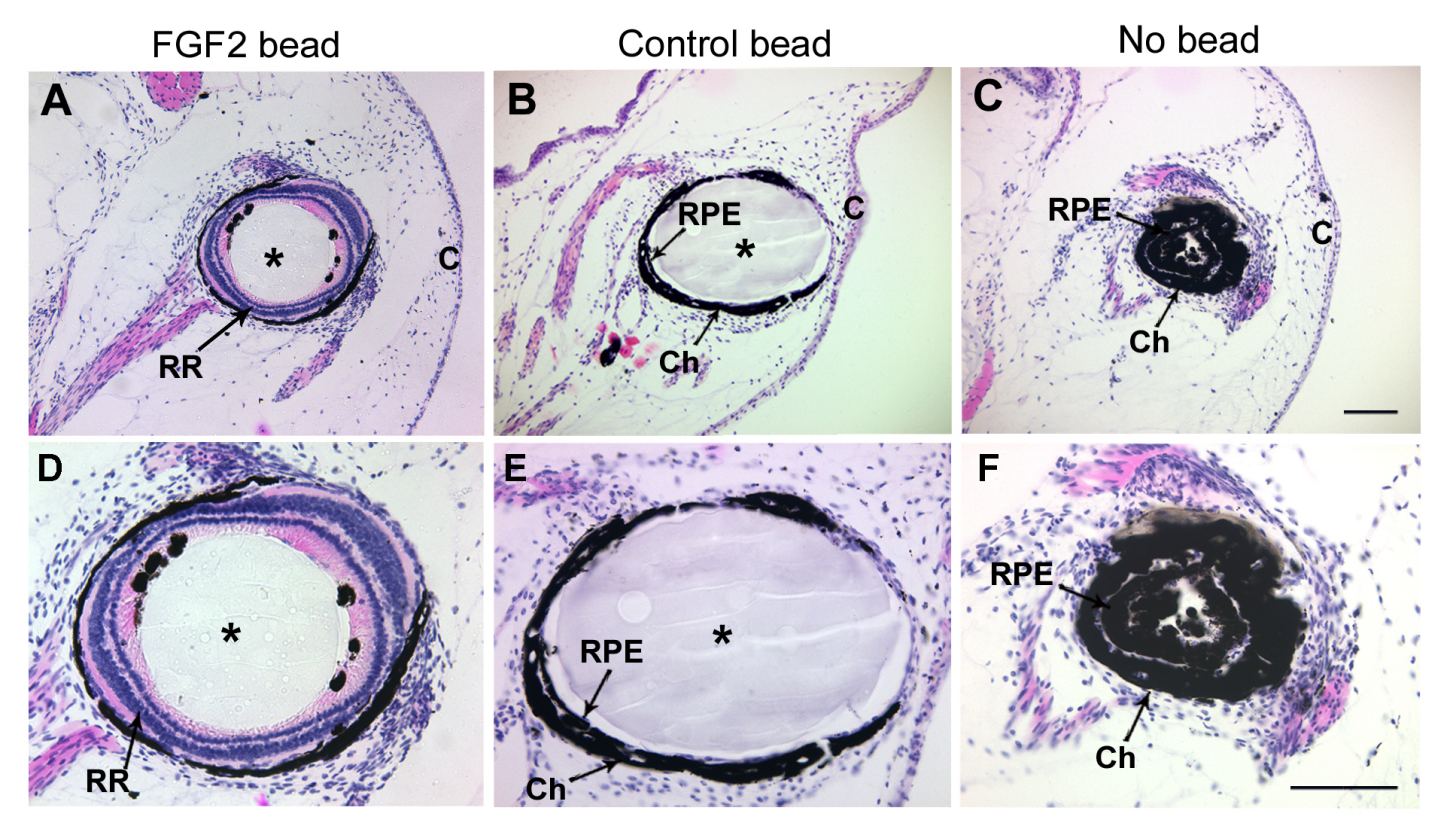
The RPE is a likely source of retinal regeneration. The anterior third of the eye was dissected out, and the neural retina was removed from the posterior eyecup of *Xenopus laevis* tadpoles, at which point either an FGF-2-soaked bead (**A**, **D**), a control bead (**B**, **E**), or no bead (**C**, **F**) was introduced in eyecups. The panel shows histological sections of eyes collected 30 days postsurgery and stained with hematoxylin and eosin. **D-F** are higher magnification images of **A**-**C** respectively. Robust retinal regeneration was observed in all eyes treated with FGF-2 (**A**, **D**), whereas there was no retinal regeneration in any case of eyes exposed to control beads (**B**, **E**) or no bead at all (**C**, **F**). Abbreviations: choroid layer (Ch); retinal pigmented epithelium (RPE); regenerated retina (RR), cornea (C). Asterisks indicate beads. Scale bars represent 100 μm (scale bar in **C** applies to **A**-**C** and scale bar in **F** applies to **D-F**).

To address the origin of the regenerated retina, we surgically dissected out the anterior third of the eye, containing the cornea, iris, lens, CB and ciliary marginal zone; we removed the retina from the remaining posterior eyecup, leaving only the RPE. Either no bead, a control bead, or an FGF-2 soaked bead was then placed in the retinectomized cup, and the tissues were collected at 30 days postsurgery for histological analysis. Interestingly, we found that in each case in which the FGF-2-soaked bead remained in the eyecup (10 cases), neural retinal regeneration was evident, suggesting that the RPE was able to transdifferentiate in vivo (Figure 4A,D). On the other hand, when either a control bead was present (6 cases) or no bead was introduced in the eye (15 cases), no retinal regeneration could be detected (Figure 4B,C,E,F). These results do not exclude the possibility that the pigmented anterior tissues could also contribute to regeneration.

### Retinal regeneration requires activation of the MAPK pathway by FGF signaling

**Figure 5:**
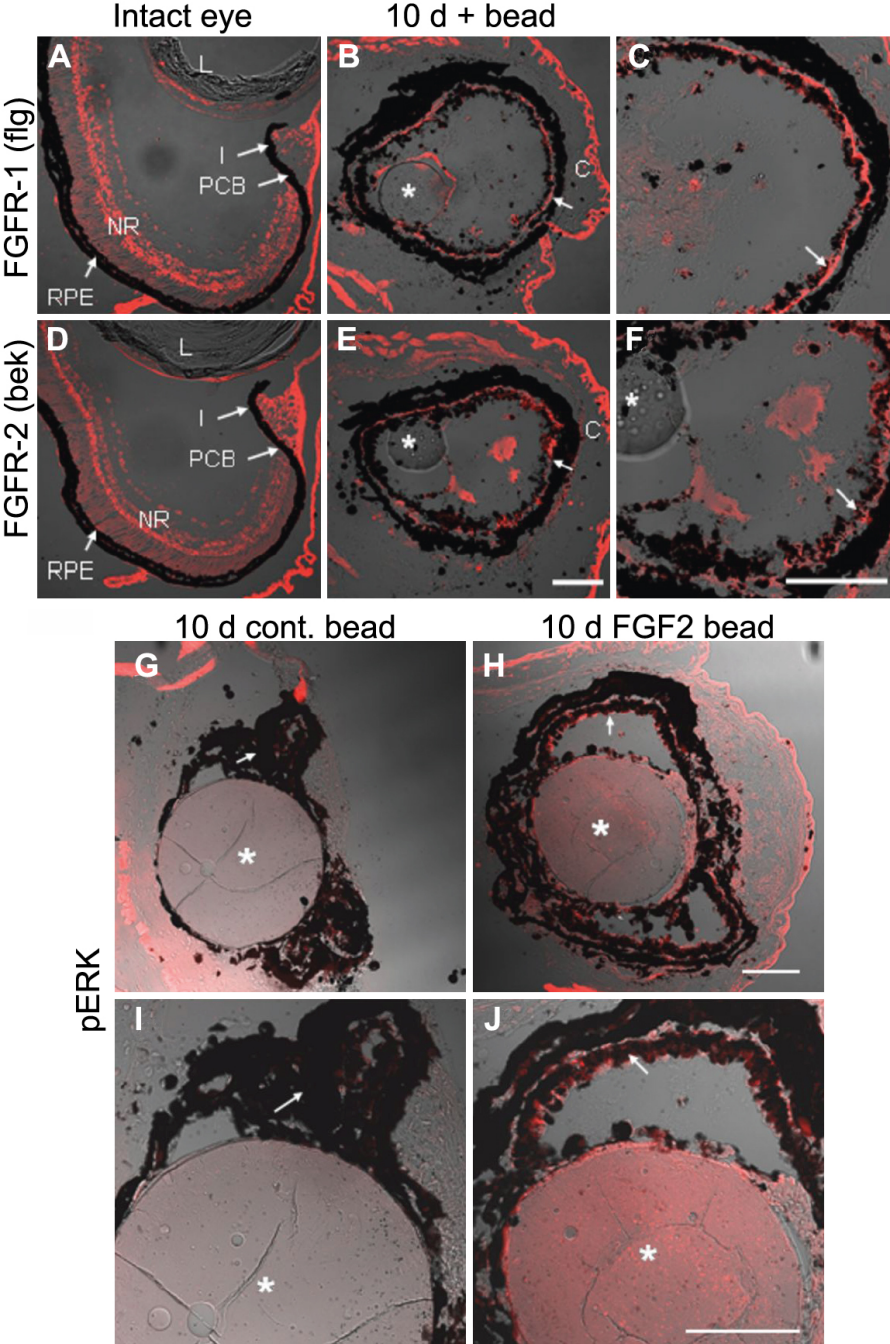
FGF receptors 1 and 2 expression and phosphorylated extracellular signal-regulated protein kinase (pERK) are upregulated during regeneration. **A**-**F**: Immunohistochemistry for FGF receptor 1 (flg, **A**-**C**) and FGF receptor 2 (bek, **D-F**) was performed on intact eyes (**A**, **D**), as well as on eyes exposed to a control bead soaked in PBS or an FGF-2 soaked bead and collected 10 days postretinectomy (**B**, **C**, **E, F**). **C** and **F** are a close up images of **B** and **E** respectively. Notice that FGF receptors (red) were detected in the neural retina and not in the pigmented tissues of the intact eye, whereas after retina removal, expression of these receptors was evident in the RPE of eyes exposed to control or FGF-2 beads (arrows). **G-J**: Immunohistochemistry for pERK (red) at 10 days postretinectomy in eyes treated with control (**G, I**) or FGF-2-soaked beads (**H, J**). **I** and **J** are a close up views of **G** and **H** respectively. Only the pigmented epithelium of eyes exposed to FGF-2 beads was labeled by the pERK antibody. Arrows point to the pigmented epithelium. Asterisks indicate control or FGF-2 soaked beads. Abbreviations: lens (L); neural retina (NR); retinal pigmented epithelium (RPE); iris (I); pigmented ciliary body (PCB); cornea (C). Scale bars represent 100 μm. Scale bar in **E** applies to **A**, **B**, **D,** and **E**; scale bar in **F** applies to **C** and **F**; scale bars in **H** and **J** apply to **G, H,** and **I, J** respectively.

Since FGF-2 is able to induce regeneration of the retina upon removal, the tissues that remain in the eye after surgery have to be able to respond to this signal through the phosphorylation of specific FGF receptors and activation of their intracellular signaling cascades. As a first step in dissecting the molecular events that take place during retinal regeneration in the *Xenopus laevis* tadpole, we decided to analyze the expression pattern of FGF receptors in the intact and regenerating eye by immunohistochemistry. Using antibodies for FGFR1 (flg) and FGFR2 (bek), we found expression of these proteins in cell membranes throughout the intact neural retina (Figures 5A,D). However, no expression of these receptors was detected in the pigmented epithelia of the intact eye. This does not mean that the receptors are completely absent from this tissue, but that their expression falls below the detection level of this technique (Figures 5A,D). Ten days after retina removal, expression of these receptors was observed indistinctly in the pigmented tissues of eyes treated with either FGF-2 or control beads (Figures 5B,E; higher magnification in Figures 5C,F). This suggests that in the absence of neural retina, the pigmented epithelium becomes responsive to FGF signaling by upregulating FGF receptors. We have not tested the expression of FGF receptors 3 and 4, which could also be regulated in this process.

Regarding intracellular signaling cascades, the MAPK pathway is probably the most common and well characterized signaling mechanism activated by receptor tyrosine kinases-like FGF receptors. It is involved in a variety of developmental processes in different tissues and model organisms, and has recently been shown to be involved in limb regeneration in *Xenopus laevis* [35]. Therefore we decided to investigate its involvement in the process of retinal regeneration in this animal model.

Upon activation by a ligand, receptor tyrosine kinases can phosphorylate Ras proteins, which in turn activate Raf. Raf can then phosphorylate MEK, which activates ERK through phosphorylation in two different residues. Activated ERK is translocated to the nucleus, where it can phosphorylate transcription factors and thus regulate gene expression [36]. We analyzed ERK phosphorylation by immunohistochemistry and found that phosphorylated ERK (pERK) is detected in eyes treated with FGF-2 but not with control beads 10 days post retinectomy (Figures 5G-J), indicating activation of this pathway by FGF-2.

**Figure 6:**
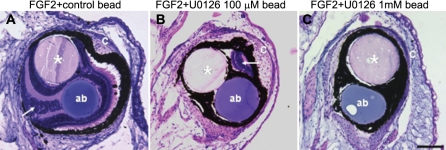
Inhibition of the MAPK pathway decreases FGF-induced retinal regeneration in *Xenopus laevis*. U0126, a potent inhibitor of MEK, was used for inhibition studies at concentrations of 100 µM and 1 mM. Tadpoles were retinectomized. Both an FGF-2-soaked heparin-coated bead and an affigel blue bead soaked in either the inhibitor or in DMSO for control were introduced in their eyes. The pictures show representative sections of eyes collected at 30 days postsurgery and stained with hematoxylin and eosin. **A**: Normal retinal regeneration was evident in the eyes that were treated with FGF-2 plus a control affigel blue bead. **B**: Eyes treated with FGF-2 and 100 µM U0126 affigel blue beads showed severe reduction of regeneration. **C**: No regeneration of the retina was detected in eyes treated with an FGF-2 bead and a 1 mM U0126 affigel blue bead. Arrows point to regenerated neural retina. Asterisks indicate FGF-2-soaked heparin beads. Abbreviations: affigel blue bead, soaked in the inhibitor or control (ab); cornea (C). Scale bar in **C** represents 100 μm and applies to all panels.

To confirm the functional significance of these results we decided to use a pharmacological inhibition approach. U0126, an inhibitor of MEK, has been widely used to inhibit this pathway in different systems including *Xenopus laevis* [35]. Stages 51–54 tadpoles were retinectomized, and both FGF-2 beads and beads soaked in different concentrations of the inhibitor (or in vehicle for control) were introduced in the eyes. The tadpoles were collected at 30 days postsurgery and processed for histological examination. Figure 6 shows that eyes treated with FGF-2 and a control bead had a normal regenerated retina (Figure 6A); regeneration was significantly reduced in eyes treated with FGF-2 plus a 100 µM U0126-soaked bead (Figure 6B, Table 1), and was absent in eyes treated with FGF-2 and a 1 mM U0126-soaked bead (Figure 6C, Table 1). Our results indicate that activation of the MAPK pathway by FGF is critical for retinal regeneration to take place in this system.

**Table 1:** Summary of the results obtained with the MAPK inhibition experiments.

**Treatment**	**Regeneration**			**n**	**Significant**	**p-value**
**None**	**Reduced**	**Normal**
FGF+control	0	3	13	16		
FGF+U0126 100 mM	0	5	5	10	yes	<0.01
FGF+U0126 1 mM	5	0	0	5	yes	< 0.001

A chi square test was used to assess statistical significance when comparing eyes treated with FGF-2 plus inhibitor (U0126) to control eyes (FGF-2 plus control bead). Treatment with the MEK inhibitor significantly reduced retinal regeneration in this system.

## Discussion

*Xenopus laevis* provides numerous advantages as an animal model for research, such as the ease of its raising and fertilization in the laboratory and the large number and accessibility for manipulation of the embryos and tadpoles. It also provides the possibility of doing transgenics and using approaches such as morpholinos and RNAi to knock down gene expression. However, its potential for retinal regeneration research has not been fully explored.

Sologub [16] and Sakaguchi et al. [17] used *Xenopus laevis* larvae or adults in RPE transplantation studies and found that the RPE has the potential to transdifferentiate if the appropriate environment or signal is present. One good candidate signaling molecule is FGF. The FGF pathway is involved in a variety of developmental and regenerative processes in different animal models, controlling cell proliferation, differentiation, and survival. Particularly in eye regeneration, this pathway is involved in the regeneration of the lens in newts and in retinal regeneration in chick embryos [9,37]. In *Xenopus laevis*, studies performed in culture, incubating RPE explants from stages 47–53 tadpoles in the presence of FGF-2 for up to 30 days, induced their transdifferentiation in vitro into different retinal neuron and glial types [17].

In vivo approaches to retinal regeneration in *Xenopus laevis* have been limited. When Mitashov and Maliovanova [23] removed the retina from the postmetamorphic *Xenopus laevis* eye, retaining only the RPE and ciliary margin, they found the retina could be partially regenerated mainly by proliferation of stem cells normally present in the ciliary margin of the eye. Transdifferentiation of the RPE did not seem to play a major role in this process. In addition, when small lessions are inflicted to the retina and adjacent RPE of postmetamorphic *Xenopus laevis*, a repair process takes place, the extent of which depends on the size of the ablation. The cellular sources of this regeneration are not clear, however the ciliary margin of the eye and intraretinal nests of proliferating cells that exist in the inner nuclear layer (INL) and outer nuclear layer (ONL) have been suggested [38]. The contribution from the RPE at this time cannot be discarded in such studies.

Most of the studies dealing with retinal regeneration in *Xenopus* are not recent, and a much thorough understanding of this process could be achieved with the use of modern tools. The latest study in which retinal ablation was attempted in vivo was performed by Yoshii et al. in postmetamorphic frogs [24]. The regenerated retina seemed to be derived from transdifferentiation of RPE cells that migrated away from their RPE layer and attached to the vascular membrane that was left behind in their surgery. This could be a promising system to study RPE transdifferentiation. It would be important in this model to further characterize the regenerated retina and to identify the molecular mechanisms involved in regeneration.

In the present study, we introduced a new model system for retinal regeneration research. We showed that *Xenopus laevis* tadpoles at stages 51–54 are able to regenerate a neural retina in vivo after complete surgical removal in the presence of an FGF-2 soaked bead. Such regeneration was not observed in eyes treated with control beads, which means that FGF plays an inductive role in this process (Figure 1). This is consistent with the aforedescribed results for in vitro studies in *Xenopus laevis*. We used molecular markers to characterize the regenerated tissue and found that, as expected, it possessed the differentiated cell types that constitute a normal retina, following the correct pattern (Figure 2, Appendix 1 and Appendix 2). This suggests that FGF treatment does not alter the proper differentiation and patterning of the retina during regeneration. In addition, we showed that the regenerated retina is in many cases able to form an optic nerve (Figure 3), something that has not been demonstrated in some other models of retinal regeneration.

The cellular sources of regeneration in this system are likely to be the RPE or the pigmented epithelia of the anterior region of the eye (pigmented ciliary body or iris), since these are the only tissues that remain within the eye after surgery. Noticeably, all of these structures have been shown to be highly plastic in other contexts. We showed that upon removal of the anterior portion of the eye, the eye was still able to regenerate a retina following FGF-2 induction, suggesting RPE involvement (Figure 4). This does not discard the possibility that the ciliary body and iris might also participate in regeneration when they are left in the eye.

We wanted to go one step further and elucidate the mechanism of induction of regeneration by FGF. MAPK signaling is an important signal transducer for FGF receptors. It has been shown to be activated by FGF during *Xenopus laevis* development and to play a role in *Xenopus laevis* limb regeneration [35,39]. MAPK signaling is also known to mediate the in vitro transdifferentiation of RPE cells to neural retina in newts [40], and retinal regeneration in the embryonic chick in vivo [9]. In the present study, we demonstrated that the MAPK pathway also plays a crucial role in retinal regeneration in our model system. We found that, upon retina removal in *Xenopus laevis* tadpoles, a fast upregulation of FGF receptors 1 and 2 occurred in the pigmented epithelia of the eye (Figure 5), and when a source of FGF-2 was placed in the eye, it activated the MAPK pathway as observed by ERK phosphorylation (Figure 5). Finally, we assessed the functional significance of MAPK activation by inhibiting the pathway at the level of MEK in the presence of exogenous FGF. We concluded that activation of this pathway was essential for regeneration to occur, since its inhibition led to a significant decrease in retinal regeneration. Interestingly, this mechanism is shared with other animal models of RPE transdifferentiation, such as the embryonic chick, which points to the similarity of this process in different animals and the likelihood of translating the findings made in one model to others. Such similarity provides the possibility of addressing the same questions by exploiting the advantages of different systems to overcome the limitations of others. We did not study the activation of other intracellular signaling cascades that can also be activated by FGF signaling, such as the PI3 Kinase pathway and PLCγ, and therefore cannot discard their involvement in this process.

In conclusion, our characterization of retinal regeneration in the *Xenopus laevis* tadpole could contribute significantly to the elucidation of the molecular mechanisms that drive retinal regeneration. We have started to do so by analyzing the role of the MAPK signaling pathway, but this is only the beginning of the possibilities that can be explored using this model.

## Appendix 1. Regenerated retina immunolabeled by Xen-1 but not by RPE-65.

To access the data, click or select the words “Appendix 1.” This will initiate the download of a compressed (pdf) archive that contains the file. Tadpole eyes were retinectomized, and an FGF-2-soaked bead (asterisk) was introduced in them. These eyes were collected at 0 (**B**, **G**), 10 (**C**, **H**), 20 (**D**, **I**), and 30 (**E**, **J**) days postsurgery and processed for immunohistochemistry. Labeling by Xen-1, a neural marker, was observed in the intact retina (**A**). Its expression was lost inside the eye at 0 and 10 days postsurgery (**B** and **C**), but was detected again in the regenerated retina at 20 and 30 days postoperation (**D** and **E**). Inset in **A** is a close up of the boxed area, showing that the ciliary marginal zone (CMZ) and the nonpigmented ciliary body (NPCB) were positive for Xen-1. Inset in **E** is a dark-field a close up of the 30-day regenerated retina. RPE-65 expression was only detected in the RPE and not in the retina or other pigmented eye tissues at any of the time points analyzed (**F**-**J**). Abbreviations: retinal pigmented epithelium (RPE); lens (L); retina (R); optic nerve (ON); pigmented ciliary body (PCB); regenerated retina (RR); cornea (C). Scale bar in **J** represents 100 μm and applies to all panels.

## Appendix 2. Cell marker distributions in INL of the regenerated retinas mimic younger retinas.

To access the data, click or select the words “Appendix 2.” This will initiate the download of a compressed (pdf) archive that contains the file. Immunolabeling for AP2α (**A**, **B**) or islet-1 (**C**, **D**) was performed on 30-day regenerated retinas of stages 51–54 tadpoles (exposed to FGF-2; **A**, **C**) and on intact retinas of stage 46 tadpoles (**B**, **D**). Notice the similar distribution of the labeled cells. Abbreviations: ganglion cell layer (GCL); inner nuclear layer (INL); outer nuclear layer (ONL). Scale bar in **D** represents 100 μm and applies to all panels.

## Appendix 3. Comparison between intact and 30-day regenerated retinas.

To access the data, click or select the words “Appendix 3.” This will initiate the download of a compressed (pdf) archive that contains the file. The cells in each cell layer were counted on 12 different 50-μm-long areas across the width of the retina in either intact or 30-day regenerated retinas, and their percentages with respect to the total number of cells in each area was determined. The length of the rod photoreceptor outer segments was also measured and compared between both types of retinas. A Student *t*-test was performed to evaluate the results, and no statistically significant differences were found between the two groups. Abbreviations: ganglion cell layer (GCL); inner nuclear layer (INL); outer nuclear layer (ONL); photoreceptor (Pr.); standard deviation (St. Dev.).
